# Immunisation with UB-312 in the Thy1SNCA mouse prevents motor performance deficits and oligomeric α-synuclein accumulation in the brain and gut

**DOI:** 10.1007/s00401-021-02381-5

**Published:** 2021-11-06

**Authors:** Jacqui T. Nimmo, Harry Smith, Chang Yi Wang, Jessica L. Teeling, James A. R. Nicoll, Ajay Verma, Jean-Cosme Dodart, Zhi Liu, Feng Lin, Roxana O. Carare

**Affiliations:** 1 Clinical Neurosciences, Clinical and Experimental Sciences, Faculty of Medicine, University of Southampton, Southampton, UK; 2 Vaxxinity, Dallas, TX USA; 3 United Biomedical, Inc, Hauppauge, NY USA; 4 United Neuroscience, Dublin, Ireland

**Keywords:** Immunotherapy, Synucleinopathies, Thy1SNCA, Alpha synuclein, Gastrointestinal pathology, Motor behaviour

## Abstract

Alpha synuclein has a key role in the pathogenesis of Parkinson’s disease (PD), Dementia with Lewy Bodies (LBD) and Multiple System Atrophy (MSA). Immunotherapies aiming at neutralising toxic αSyn species are being investigated in the clinic as potential disease modifying therapies for PD and other synucleinopathies. In this study, the effects of active immunisation against αSyn with the UB-312 vaccine were investigated in the Thy1SNCA/15 mouse model of PD. Young transgenic and wild-type mice received an immunisation regimen over a period of 6 weeks, then observed for an additional 9 weeks. Behavioural assessment was conducted before immunisation and at 15 weeks after the first dose. UB-312 immunisation prevented the development of motor impairment in the wire test and challenging beam test, which was associated with reduced levels of αSyn oligomers in the cerebral cortex, hippocampus and striatum of Thy1SNCA/15 mice. UB-312 immunotherapy resulted in a significant reduction of theαSyn load in the colon, accompanied by a reduction in enteric glial cell reactivity in the colonic ganglia. Our results demonstrate that immunisation with UB-312 prevents functional deficits and both central and peripheral pathology in Thy1SNCA/15 mice.

**Supplementary Information:**

The online version contains supplementary material available at 10.1007/s00401-021-02381-5.

## Introduction

Synucleinopathies are chronic progressive neurodegenerative diseases that are characterised by accumulation of alpha-synuclein (αSyn) in the brain. The synucleinopathies include Parkinson's disease (PD), Parkinson’s disease Dementia (PDD), dementia with Lewy bodies (DLB) and Multiple systems atrophy (MSA) [40]. Several treatment options are available for these diseases, but they only provide symptomatic relief and do not directly target the underlying pathology. With the current aging population, PD and DLB cases are escalating, highlighting the urgent need for developing therapies that can prevent or delay the progression of neurodegeneration. Anti-αSyn immunotherapy aims to achieve this by targeting and neutralising toxic proteins that have been released from cells and prevent their propagation to neighbouring cells.

αSyn is predominantly an intracellular protein, thus potentially limiting the effects of immunotherapy. However, a number of studies have demonstrated that extracellular αSyn also plays a role in disease and is involved in the propagation of αSyn between neurons. αSyn can be detected in cerebrospinal fluid (CSF) as well as in the interstitial fluid (ISF) of the brain parenchyma [16]. αSyn is mainly secreted from cells by exocytosis [21], or directly released into the extracellular space due to cell lysis and death. Internalisation of the protein by neighbouring cells results in the formation of protein aggregates, which could result in the propagation of the disease to anatomically connected brain regions [10, 25]. This mechanism has been demonstrated in cell culture studies in which addition of αSyn preformed fibrils to primary neuronal cultures, at concentrations comparative to those found in CSF (0.1 ng/ml), induced endogenous αSyn to form LB-like inclusions. This did not occur with monomeric αSyn, which is consistent with oligomeric and fibrillary αSyn species being the toxic species in PD [55]. Immunotherapy, therefore, provides an opportunity for prevention of the propagation of αSyn by sequestering and/or clearing toxic extracellular αSyn species.

A diagnosis of PD involves characteristic changes in motor function including symptoms of bradykinesia, rigidity and tremor. Immunotherapy trials in Alzheimer’s disease have shown more progress towards clinical benefit at the earliest disease stages [30], highlighting the necessity for early intervention with immunotherapy. In PD, motor symptoms are typically preceded by non-motor, or autonomic dysfunction by up to 20 years [15, 42, 65]. One of the most prevalent non-motor features of PD is gastrointestinal (GI) dysfunction [17, 37]. A number of independent longitudinal population-based studies on patients that go on to develop PD disease have shown that over 50% of PD subjects suffer GI dysfunction [32]. Of note, one large UK based population study involving over 8100 subjects with PD and 46,700 without PD found that 10 years prior to the onset of Parkinsonism, the incidence of GI dysfunction was 6% higher in PD subjects compared to controls [46]. A meta-analysis of early non-motor features highlighted that the strongest non-motor associations with PD are gastrointestinal (GI) complications, which double the risk of developing PD [32, 37, 51]. This has led to the hypothesis that the GI tract is an initiator site for PD pathology and opens a window for therapeutic intervention at very early stages of the disease. GI dysfunction could be attributed to αSyn pathology in the dorsal motor nucleus of the vagus (DMV) and/or in the enteric nervous system (ENS). Whether αSyn pathology commences in the GI tract or brain has not been established, with conflicting reports supporting both hypotheses [27]. Despite this, a number of neuropathological studies in colonic biopsies have correlated GI dysfunction with the presence of αSyn aggregates in the GI tract suggesting that targeting αSyn may alleviate GI dysfunction [22, 43, 52, 57, 63]. GI inflammation with increased levels pro-inflammatory cytokines has also been reported in colonic biopsies from PD patients [13]. A number of factors have been found to contribute to GI inflammation including gut permeability, microbiota, enteric glial cells (EGCs), as well as αSyn. EGCs are located mainly in the myenteric and submucosal compartments and are akin to astrocytes of the CNS as they provide structural and trophic support to enteric neurons, maintain homeostasis, contribute to the blood-enteric-barrier, undergoing reactive gliosis in response to injury, with increased expression of GFAP [4, 9]. The ability to target αSyn accumulation at early stages in the development of disease would hold more promise for preventative treatments. The effect of immunotherapy on GI pathology has not yet been investigated to our knowledge, and this could provide an efficacious treatment strategy for PD before too much damage to the brain has occurred.

Immunotherapy is a rapidly developing area of therapeutics with the rise of next-generation technology enabling the design and manufacture of highly targeted vaccines for neurodegenerative disease-associated proteins. Very few immunotherapies have been developed for synucleinopathies which have been extensively reviewed in [35, 47], and of which only two active immunotherapies are in clinical trials. One of these is UB-312 designed by Vaxxinity based on UBITh technology, which has been found to elicit an enhanced B-cell response while avoiding harmful pro-inflammatory T-cell responses [36, 58, 59]. The UB-312 peptide was selected from screening over 60 B-cell epitopes of αSyn for immunogenicity in guinea pigs [36]. UB-312 incorporates a 10 residue long, fully synthetic, peptide from the C terminal domain (CTD) of αSyn which was mixed with an adjuvant composed of polyanionic Cytosine phosphoguanine (CpG) oligodeoxynucleotide (ODN) and Adju-Phos. Intrinsic self T cell epitopes are replaced by foreign un-selective UBITh T helper peptides that are covalently linked to the functional antigenic αSyn peptides [58, 59]. This increases the immunogenicity of UB-312 to ensure a robust and targeted response against αSyn, and not the carrier protein. Antibodies generated from UB-312 immunisation in guinea pigs were found to have high specificity for αSyn oligomers and fibrils and also demonstrated specific recognition of pathological forms of αSyn aggregates in post mortem brains of different synucleinopathies [36]. UB-312 is currently in a phase 1 trial to assess safety and tolerability of vaccination in healthy and mild PD patients (NCT04075318).

This study aims to investigate the effects of UB-312 immunisation on the functional outcome and neuropathology in Thy1SNCA/15 mice. Furthermore, this study aims to investigate whether UB-312 immunotherapy has an effect on gastrointestinal pathology in order to assess its potential in the treatment of prodromal PD cases that manifest early GI complications.

## Materials and methods

### Animals

Thy1SNCA/15 mice (Stock No: 017682) were obtained from the Jackson Laboratory (Bar Harbour, Maine, USA) and rederived at the University of Southampton to establish and maintain colonies. The Thy1SNCA/15 mice overexpress 1–2 copies of the gene encoding human wild-type αSyn that is driven by the mouse thymus cell antigen 1 (Thy1) promoter leading to neuronal expression of αSyn in the brain and gut [7, 8, 60]. Thy1SNCA/15 mice were first described by Choi et al. and demonstrated widespread αSyn expression with no reported LB-like aggregates or phosphorylated αSyn up to 10 months of age [8, 44]. We hypothesise that the overexpression of normal human αSyn present in Thy1SNCA/15 and not aberrant amounts of mutant αSyn, represent a suitable model for the study of early stages of aSyn toxicity. The closely related Thy1SNCA/61 mouse model has demonstrated significant motor impairment in a number of behavioural tests as early as 2 months of age; however, to our knowledge this is the first time that behavioural deficits have been characterised in the Thy1SNCA/15 mice.

All mice were housed in groups of 5–10, kept under a standard 12-h light/dark cycle and fed a standard RM1 chow diet (SDS, UK) and water ad libitum. All procedures were carried out in accordance with animal care guidelines stipulated by the United Kingdom Animals (Scientific Procedures) Act 1986, Home Office licence.

### Vaccination of mice with UB-312 and antibody titres

**Fig. 1:**
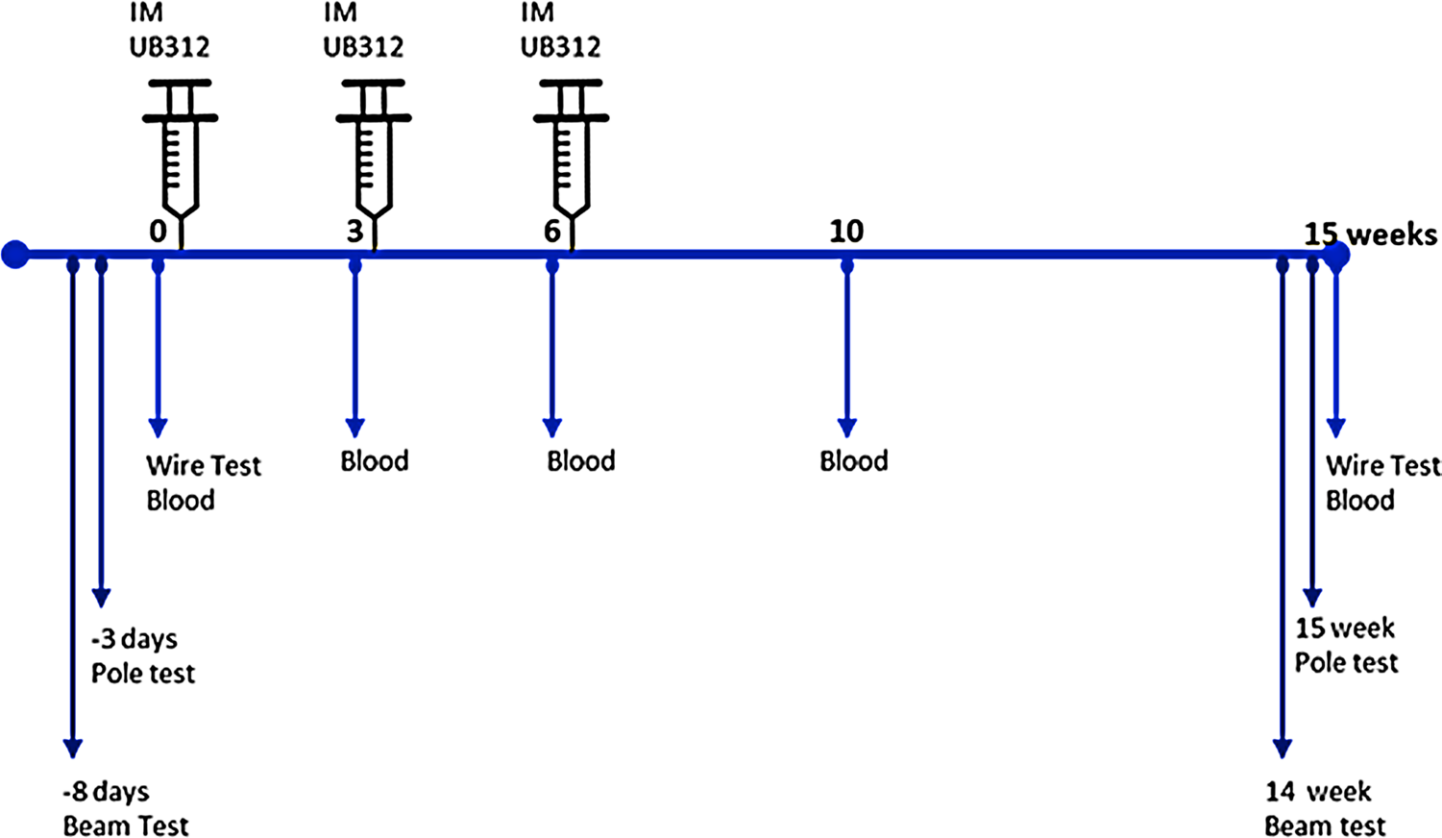
Schematic representation of immunisation regime. 10-week-old Thy1SNCA/15 mice and Wt controls were administered intramuscular injections of either UB-312 or adjuvant 3 weeks apart. Blood samples were collected for antibody titre analysis prior to each injection, on week 10 and week 15 (terminal time point). Behaviour tests were performed prior to the initiation of immunotherapy and at the end of the 15 week study period

There is evidence that immunotherapy is more efficient and with fewer side effects when administered early [20, 35].The immunisation regime for the present study is summarised in Fig. 1. 10-week-old Thy1SNCA/15 mice were administered three intramuscular injections (3 weeks apart) of either UB-312 (40 µg per injection, *n* = 29) or the adjuvant (Adju-Phos^®^ and CpG1) (*n* = 27). 10-week-old non-transgenic C57BL/6J littermates also received equivalent immunisations with adjuvant (*n* = 22). The UBITh vaccine technology drives a specific and selective humoral response that requires several doses (a priming regimen of three immunisations) to induce a strong antibody response. Moreover, UBITh vaccines are designed so that the immune system will not recognize the endogenous protein as an antigen, resulting in a decrease of antibody titres overtime in the absence of the peptide immunogen (vaccine). Thus, regular boosts are necessary to maintain sustained antibody titres.

Sera were collected before each injection, and at weeks 10 and 15 after the primary injection for antibody titre analysis. Antibody titres were measured using an anti-αSyn enzyme immunoassay (EIA) kit (United Biomedical, INC.) following instructions from the manufacturers. The kit employs a synthetic target peptide immunosorbent against the region K97-D135 of alpha synuclein. Briefly, serum samples were serially diluted in proprietary specimen diluent to reach dilutions of 1:100, 1:1000, 1:10,000, and 1:100,000. Inactivated guinea pig serum was used as a negative control, and inactivated guinea pig serum that contained a high titre of anti-αSyn antibodies (specific for the K97-D135 αSyn peptide) was used as a positive control. The serum dilutions and controls were added to the reaction microplate and incubated at 37 °C for 1 h. After washing, the reaction plate was then incubated at 37 °C for 30 min in horseradish peroxidase-conjugated recombinant protein A/G. The reaction plate was washed and incubated at 37 °C for 15 min in proprietary 3,3′,5,5′-tetramethylbenzidine (TMB) working solution. 1.0 M sulphuric acid was added to terminated the reaction. The absorbance of each well was measured at 450 nm on a FLUOstar Optima microplate reader. Antibody titres were calculated using GraphPad Prism software as and presented as log OD.

Fifteen weeks after the prime injection, mice were terminally anaesthetised with pentobarbitone (200 mg/kg) and perfused for immunohistochemistry (Tg-UB-312, *n* = 12; Tg-Adj, *n* = 11; WT-Adj, *n* = 9) or biochemical analysis (Tg-UB-312, *n* = 17; Tg-Adj, *n* = 16; WT-Adj, *n* = 13). For immunohistochemical analysis, mice were intracardially perfused with PBS (0.01 M) followed by 4% Paraformaldehyde (PFA) (in 0.01 M PBS, pH 7.4). The brains and intestines (duodenum and proximal colon) were dissected out and submersed in 4% PFA for a further 4 h, and subsequently transferred to 30% sucrose for cryoprotection. For western blot analysis, mice were perfused with ice-cold PBS (0.01 M) and the cortex, hippocampus, and striatum were immediately dissected out on ice cold PBS and snap-frozen on dry ice for further processing.

### Behaviour testing

Prior to immunisation and at 15 weeks after the first immunisation dose, mice were subject to three different behavioural tests, each performed on separate days including habituation periods such that there was no overlap of behaviour tests on any day. The order of the tests and habituation periods were kept the same before and after treatment (Tg-UB-312, *n* = 29; Tg-Adj, *n* = 27; WT-Adj, *n* = 22). The assessor was blinded to the animal’s treatment status.

#### Challenging beam traversal test

Mice were trained to traverse a 1 m-long beam composed of 4 equal segments that become narrower towards the end (3.5, 2.5, 1.5, 0.5 cm width). Mice were placed on the wide end of the beam and encouraged to traverse the beam to a clean cage on the opposite side. Mice were given five trials per day over 3 days followed by a test day. On the test day, a 1 cm^2^ wire mesh was placed over the beam segments and the mice were allowed to freely traverse the beam for five trials. Video recordings were analysed for each trial and the number of errors were recorded. An error was considered if the mouse was moving forward and one of their feet slipped halfway down the wire mesh. The average number of errors over the five trials was calculated.

#### Pole test

The pole test consists of a vertical pole (1.5 cm in diameter and 55 cm high) secured in a clean cage. Mice were placed with their head oriented upward on the side of the pole and the times to reorient themselves 180° facing down and descend the pole were recorded. Each mouse underwent 3 days of habituation, up to five trials per session followed by a test day.

#### Wire hanging test

Mice were only subject to one trial on the wire test before and after immunotherapy. Mice were placed hanging upside down on a 6 mm-thick wire loop (20 cm in diameter) that could freely rotate on a pivot. Inappropriate behaviour such as balancing on top of the wire, or deliberate jumping off the wire was discouraged and the trial discarded or repeated. The total time to fall off the wire was recorded with a cut-off of 5 min.

### Immunohistochemistry

Sagittal sections of 20 µm thickness from brain (1800 µm from midline) or intestines were cut using a Leica Cryostat. αSyn was detected using immunofluorescence. Briefly, tissue sections were rehydrated in 0.01 M PBS (Sigma, 1002795531) and blocked in 15% normal goat serum (Fisher Scientific, 1002817944) for 1 h. The sections were incubated overnight at 4 °C in the anti-αSyn antibody, MJFR1 (1:2000, Abcam, ab138501) in 0.01 M PBS, 0.1% Triton X [1001466726, ThermoFisher]. The sections were then incubated at room temperature (RT) in an Alexa-Fluor 555 conjugated goat-anti-rabbit secondary antibody (Molecular Probes life technologies). Sections were counterstained with DAPI and mounted in Mowiol and Citifluor (ThermoFisher).

To analyse the inflammatory status in the brain and gut, markers for astrocytes (GFAP, 1:400, Dako,), microglia (Iba1, 1:400, Wako, 019-19741), T-cells (CD3 (KT3), 1:200, BioRad, MCA500G), and endothelial activation (ICAM1, 1:200, Bioledgend, 116101) were selected. Endogenous peroxidase activity was quenched with 3% H_2_O_2_ (H1009-500 ml, Sigma Aldrich) for 10 min. Heat induced antigen retrieval was performed for Iba1 staining by heating the tissue in citrate buffer (15 mM Tris sodium citrate [101578237, Sigma Aldrich], 0.1% tween, pH 6 [P1379, Sigma Aldrich]) using a Panasonic 800 W microwave at medium heat for 25 min. Non-specific binding sites were blocked with 15% normal goat serum (Fisher Scientific) for 1 h. The tissue was then incubated overnight at 4 °C with primary antibody in 0.01 M PBS, 0.1% triton X. The tissue was then incubated for 1 h in biotinylated secondary antibodies at RT. Tissue was incubated in Avidin biotin complex (ABC) for 1 h at RT (PK-6100 Vectastain ABC kit). Development of the chromogen was performed using Nickel DAB. Prior to mounting in Distyrene Plasticizer Xylene (DPX, 12658646 Fisher Scientific), the tissue was dehydrated for 2 min each in IMS 50%, 70%, 95%, 100%, counterstained with eosin and incubated in Xylene for 5 min.

### Western blot

Tissue samples were homogenised on ice using a Kontes pellet pestle homogeniser in 10% W/V Radioimmunoprecipitation assay (RIPA) buffer (ThermoFisher, 89901) with HALT protease and phosphatase inhibitor cocktail (ThermoScientific, 78442). The homogenate was centrifuged at 14,000 rpm, 4 °C in an Eppendorf 5417 R benchtop centrifuge. The pellet was discarded and the supernatant retained for analysis. The protein concentration of each supernatant was determined using Pierce bovine serum albumin (BSA) assay kit (ThermoFisher, 23227) following the manufacturer’s instructions.

A Mini-PROTEIN Tetra vertical electrophoresis cell (BioRad; 1568004) was used for the separation of protein from brain homogenates. 1 mm-thick polyacrylamide gels were prepared for either denaturing conditions or native conditions.

For Native poly acrylamide gel electrophoresis (PAGE), brain homogenates were diluted in 4X Laemmli sample buffer (BioRad, 1620112) and 20 µg protein loaded into a 10 or 12% native gel. Pure monomeric αSyn (Online resource 1 B) was run alongside the brain homogenate as a molecular weight marker (kind donation of in-house synthesised product from Prof Jessica Teeling). Protein concentrations for loading were determined from the linear range of the antibodies used (Online resource 1A). Electrophoresis was conducted at 100–150 V in Laemmli buffer (192 mM Glycine [Sigma Aldrich, G8898], 25 mM Tris Base [ThermoFisher, 10103203]) for 2 h. Semi-dry transfer was conducted using Trans Blot turbo system (BioRad, 1704150) and Mini transfer kit (BioRad, 1704270). Protein was transferred to 0.2 µm nitrocellulose membranes at 2.5 V, 2 A and 15 min. Membranes were blocked with 3% Bovine serum albumin (BSA) (Sigma Aldrich, 102052095) for 1 h at RT. After washing the membranes 3 × 5 min in Tris buffered saline (TBS) (0.25 M Tris Base, 1.5 M NaCl, pH 7.2), 0.1% tween20 (Sigma, P1379), they were incubated overnight at 4 °C in MJFR1 (1:5000; ab138501, Abcam). Revert 700 total protein stain (LiCor, 926–11015) was applied prior to blocking in BSA for normalisation of protein loading.

### Image analysis and statistics

Immunoblots were imaged on a LiCor Odyssey Fc scanner and analysed using Image Studio Lite V5.2. Immunoreactive αSyn bands were normalised to GAPDH for SDS-PAGE, and Revert for native PAGE. Immunostained tissue sections processed for fluorescence microscopy were visualised, and images captured, at 20 × using an SP8 confocal-laser scanning microscope (Milton Keys, UK). DAB immunostained tissue sections were scanned for analysis at × 20 using an Olympus VS110 high throughput Virtual Microscopy System. Images (each 0.16mm^2^) were captured from the scanned image using *Olympus VS software*. For each marker studied, the percentage area of immunoreactivity over two consecutive sections per animal was calculated using FIJI software. The average percentage area was calculated for each brain region and statistical analysis was conducted using GraphPad Prism software. A two-way analysis of variance (ANOVA) was used for behavioural analysis with Bonferroni corrections for post hoc multiple comparisons. *T*-tests were conducted unless otherwise specified for analysing αSyn immunoreactivity in western blots and immunohistochemistry. One-way ANOVA was used for analysing inflammatory markers. Post hoc analysis was conducted with Bonferroni corrections for multiple comparison analysis where applicable. Differences were considered as significant when *p*  <  0.05. Numbers (*n*) refer to the number of mice used for each experiment.

## Results

### Antibody titres

**Fig. 2:**
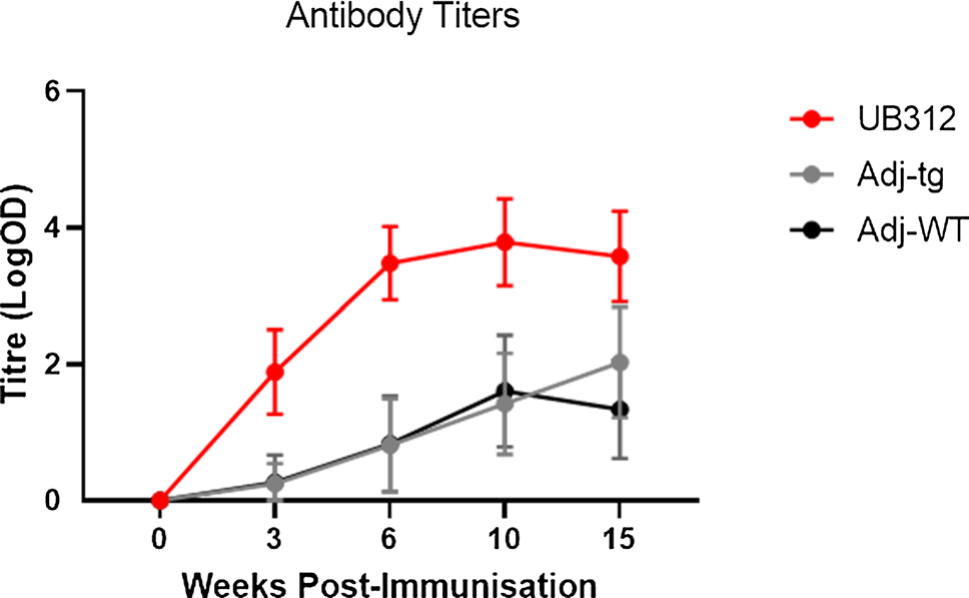
Antibody titre analysis. 10-week-old Thy1SNCA/15 mice and wild-type littermates were administered three intramuscular injections of either UB-312 or adjuvant 3 weeks apart. Blood samples were collected prior to each injection, on week 9 and week 15 (terminal time point) and antibody titres were measured for each of the collected sera. Data points represent mean ± 95% CI

All transgenic mice produced high levels of anti-αSyn97-135 antibody titres after the first injection. Titre levels rose rapidly in the first 6 weeks, peaked between weeks 6 and 10 and remained stable for the rest of the 15-week study period (Fig. 2). Unexpectedly, some Wt and Transgenic mice administered adjuvant also produced background antibody titres, but these were 2–3 orders of magnitude lower than UB-312 induced titres. The cause of this is not clear and the detection of antibody titres was 2–3 orders of magnitude lower than UB-312 induced antibody titres. Since this was observed at comparable levels in both the Wt and Thy1SNCA/15 adjuvant treated mice, it is unlikely to be a response of the rodent immune system to the human αSyn transgene. One explanation is a possible non-specific binding of anti-CpG antibodies to the substrate during the EIA analysis.

### UB-312 immunisation improves motor performance

**Fig. 3:**
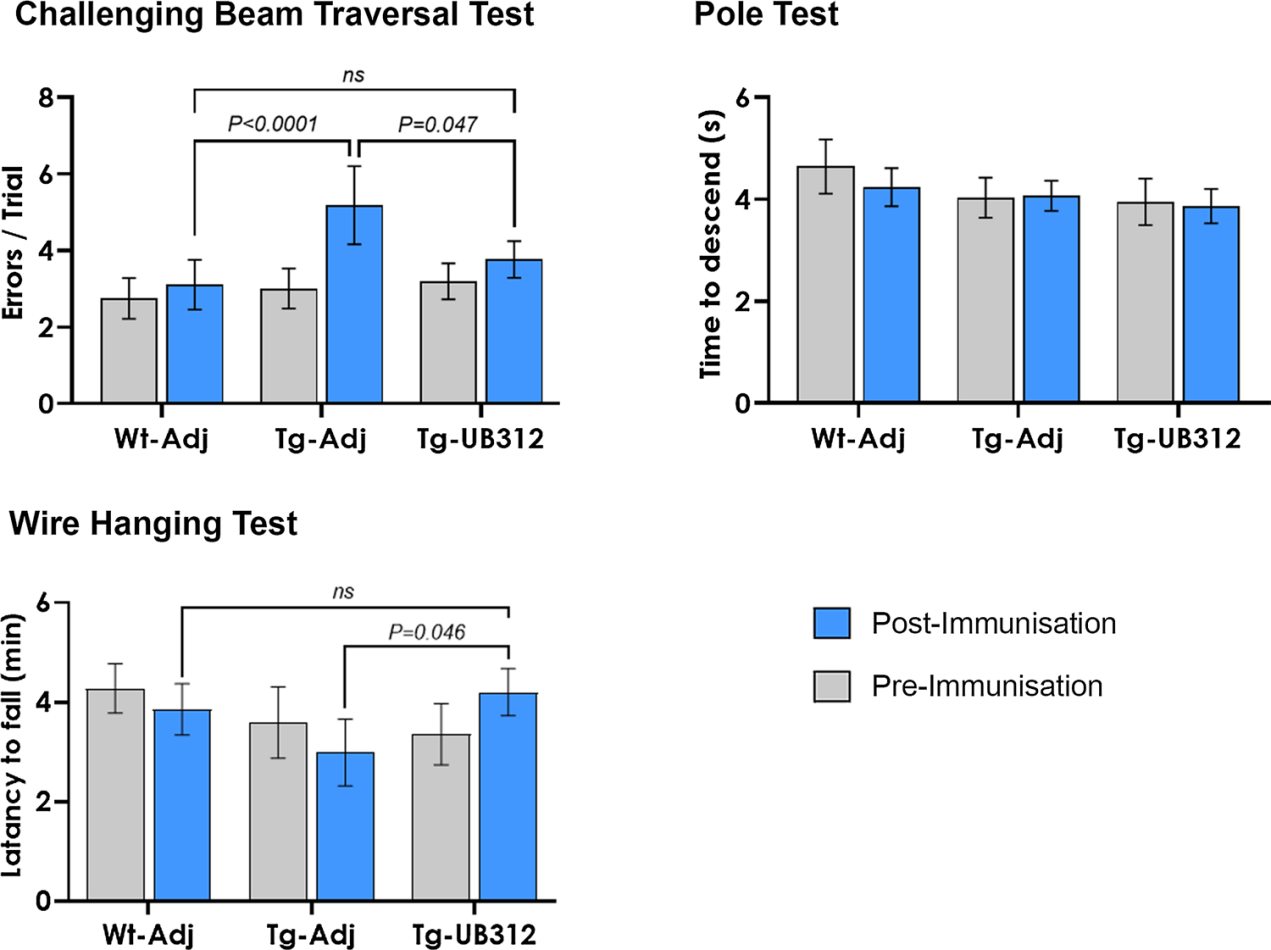
Motor behaviour analysis. 10-week-old Thy1SNCA/15 mice and wild type littermates were subject to three different motor performance tests before immunisation (Pre-immunisation) and 15 weeks after the first injection (Post-immunisation). These included the challenging beam traversal test, pole test and wire hanging test. Bars represent mean ± 95% CI

The effect of UB-312 immunotherapy on functional outcome in Thy1SNCA/15 mice was investigated using three behavioural tests designed to assess motor function. These included the wire-hanging test to measure grip strength, the challenging beam test for sensorimotor performance and the pole test for voluntary motor control [19]. At 10 weeks of age, prior to the commencement of immunotherapy, Thy1SNCA/15 mice did not show any difference in motor performance compared to Wt mice in any of the tests, as shown in Fig. 3. The motor performance of Thy1SNCA/15 mice deteriorated with age in the beam and wire test (26 weeks of age) and this deterioration was prevented with 15 weeks of UB-312 immunotherapy.

In the challenging beam traversal test, two-way ANOVA revealed a significant effect of age (*F*_(1,83)_ = 22.46, *p*  <  0.0001) and treatment (*F*_(2,83)_ = 5.72, *p* = 0.0047) on the number of foot errors. Post hoc analysis of multiple comparisons showed that the control group of Thy1SNCA/15 mice receiving adjuvant made significantly more errors per trial at 6 months of age compared to 10 weeks (*p*  <  0.0001), and compared to 6 month-old Wt mice (Wt-Adj: 3.1, Tg-Adj: 5.1; *p*  <  0.0001). The number of errors per trial was not significantly different between Wt mice and UB-312 treated Thy1SNCA/15 mice (*p* = 0.38).

In the wire hanging test, a significant effect of treatment on the age-related decline seen in the in Thy1SNCA/15 mice receiving adjuvant was observed (*F*_(2,80)_ = 4.03, *p* = 0.022). Post hoc analysis indicated a trend in reduced latency to fall time in the control group of adjuvant-treated Thy1SNCA/15 mice compared to Wt mice (Wt-Adj: 3.85, Tg-Adj: 2.98; *p* = 0.102). This was significantly lower than UB-312 treated Thy1SNCA/15 mice (Tg-Adj: 2.98, Tg-UB-312: 4.2; *p* = 0.0095). There was no significant difference between Wt mice and UB-312 treated Thy1SNCA/15 mice at the end of the treatment period (Wt-Adj: 3.85, Tg-UB-312: 4.20; *p*  >  0.99).

For the pole test, the time taken for mice to perform a turn and descend the pole was similar between Thy1SNCA/15 mice and Wt mice with no effect of age (*F*_(1,64)_ = 0.156, *p* = 0.6941) or treatment (*F*_(2,64)_ = 2.688, *p* = 0.076) on motor performance.

### UB-312 reduces αSyn oligomers in the brain

**Fig. 4:**
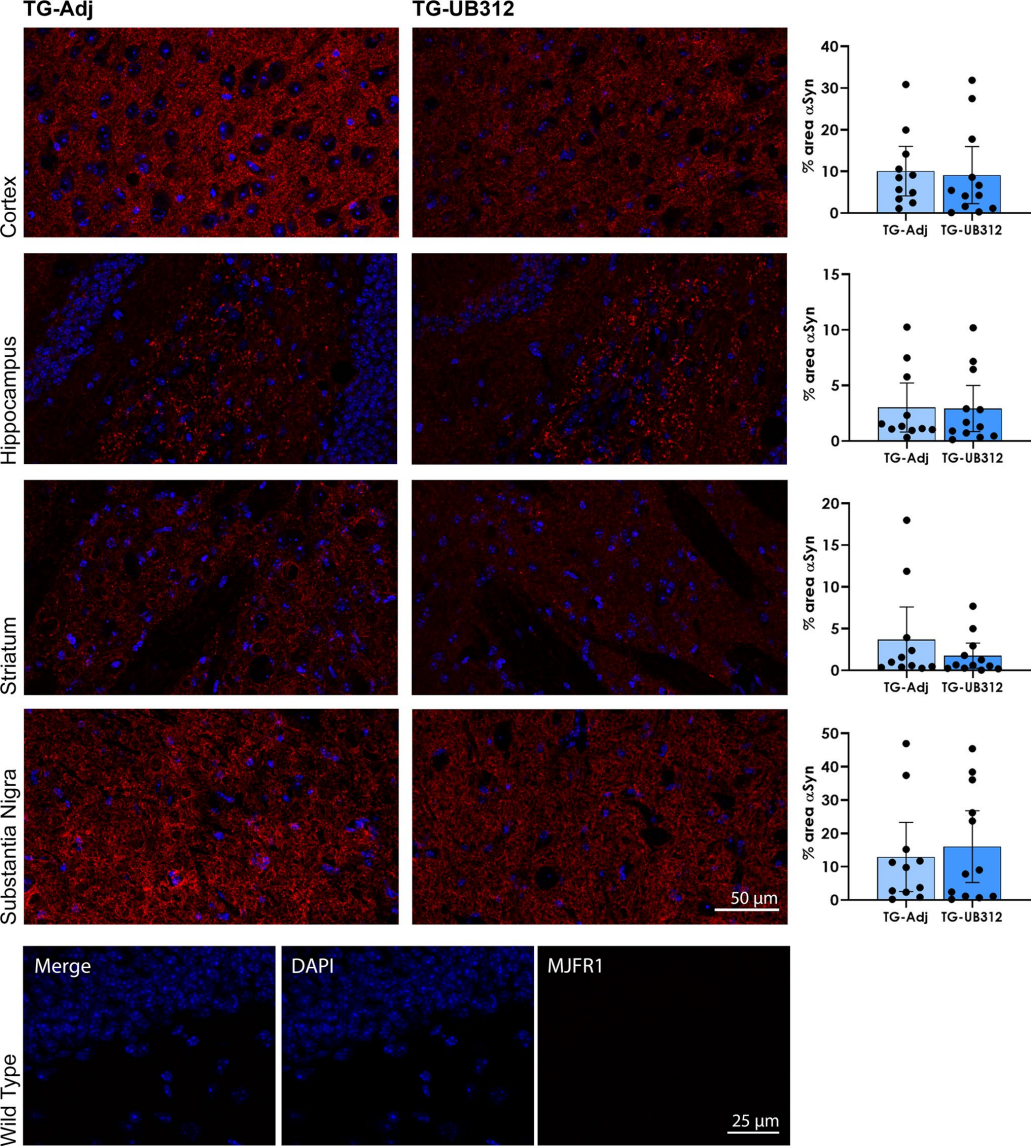
Immunohistochemistry for αSyn. αSyn immunoreactivity (Red) was quantified in the cortex, hippocampus, striatum and substantia nigra of Thy1SNCA/15 mice that received UB-312 (*n* = 12) or adjuvant (*n* = 11). Two-tailed *T* test showed no difference in the mean percentage area of αSyn immunoreactivity between treatment groups. Scale bar = 50 µm

**Fig. 5:**
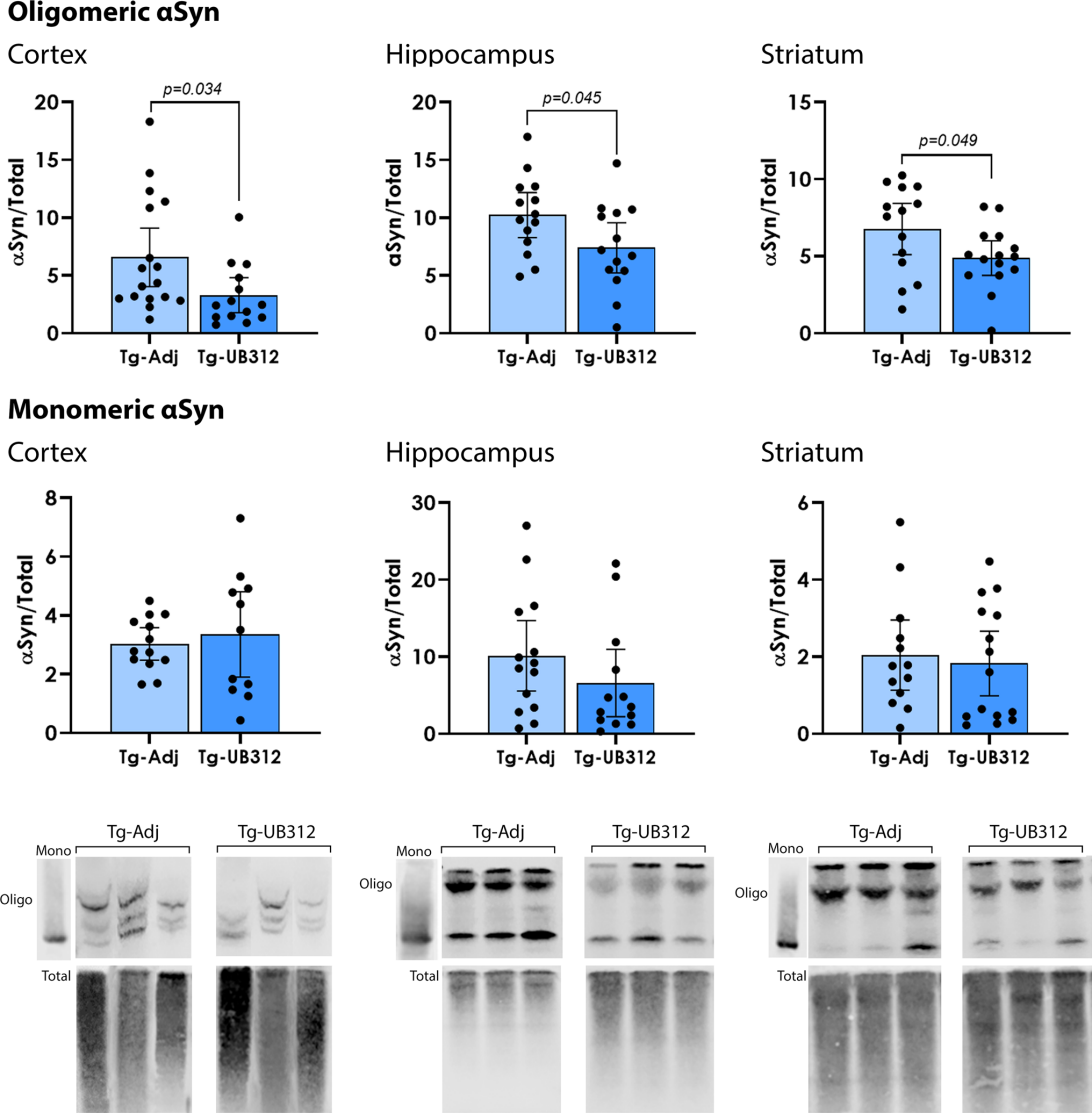
Western blot analysis of oligomeric αSyn. αSyn assemblies in brain homogenates from the cortex, striatum and hippocampus of Thy1SNCA/15 mice that had received UB-312 (*n* = 14) or adjuvant (*n* = 16) were separated by native western blot. Quantification of oligomeric and monomeric immunoreactive bands showed a significant decrease in αSyn oligomers with UB-312 treatment but not monomers. Bars represent mean ± 95% CI

At completion of the 15-week treatment period, 6-month-old mice were anesthetised and tissues collected to assess the effects of UB-312 immunotherapy on αSyn-mediated pathology. αSyn pathology was analysed by immunohistochemistry and western blot using an MJFR1 anti-αSyn antibody, which is specific for human αSyn overexpressed by the Thy1SNCA/15 mice. As expected, Wt mice showed no immunoreactivity for human αSyn and were not included in the quantitative analysis. In Thy1SNCA/15 mice, immunohistochemical staining of brain sections for αSyn showed a widespread granular or punctate pattern in grey matter, consistent with a synaptic location, although this would need validation with a synaptic marker. αSyn inclusions such as Lewy Bodies could not be detected in the brain of 6-month-old Thy1SNCA/15 mice. Quantitative analysis of the percentage area covered by αSyn immunoreactivity in each region of interest (cortex, striatum, hippocampus, substantia nigra, cerebellum; Fig. 4) did not show any difference between UB-312 and adjuvant treated mice. In order to investigate whether UB-312 specifically reduced higher molecular weight αSyn oligomers, native non-denaturing western blots were performed. Pure monomeric αSyn (produced in-house and kindly donated by Prof Jessica Teeling) was used as a molecular weight marker, and corresponded to the lowest band in the gels. The results are presented in Fig. 5 and show that UB-312 significantly reduced αSyn oligomers but not monomers in the hippocampus by 27.8% (*p* = 0.049), striatum by 27.9% (*p* = 0.045) and the cortex by 49.8% (*p* = 0.035) of Thy1SNCA/15 mice compared to control Thy1SNCA/15 mice administered adjuvant.

### UB-312 does not induce widespread astrocyte reaction

**Fig. 6:**
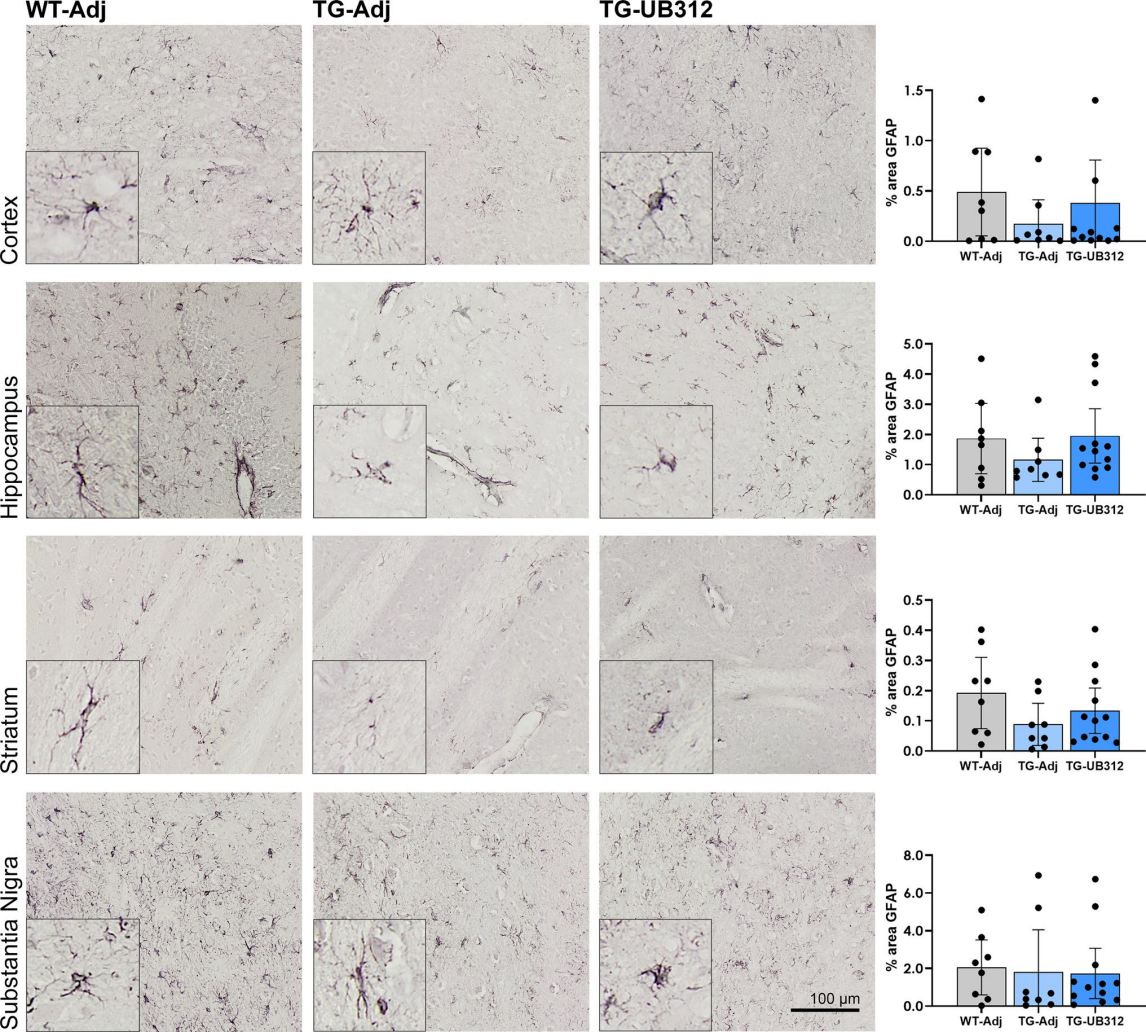
Immunohistochemistry for astrocytes. GFAP immunoreactivity was quantified in the cortex, hippocampus, striatum and substantia nigra of Thy1SNCA/15 mice that received UB-312 (*n* = 11) or adjuvant (*n* = 9) or Wt littermates that received adjuvant (*n* = 8). One-way ANOVA showed no difference between treatment groups in each brain region. Bars represent mean ± 95% CI

Immunohistochemistry was performed for the astrocyte marker, GFAP. Figure 6 shows representative images of GFAP immunoreactivity in each brain region (cortex, Hippocampus, Striatum, Substantia nigra). GFAP immunoreactivity was comparable between all three groups, which was confirmed by statistical analysis using a one-way ANOVA.

### UB-312 induces upregulation of CD64 on microglia

**Fig. 7:**
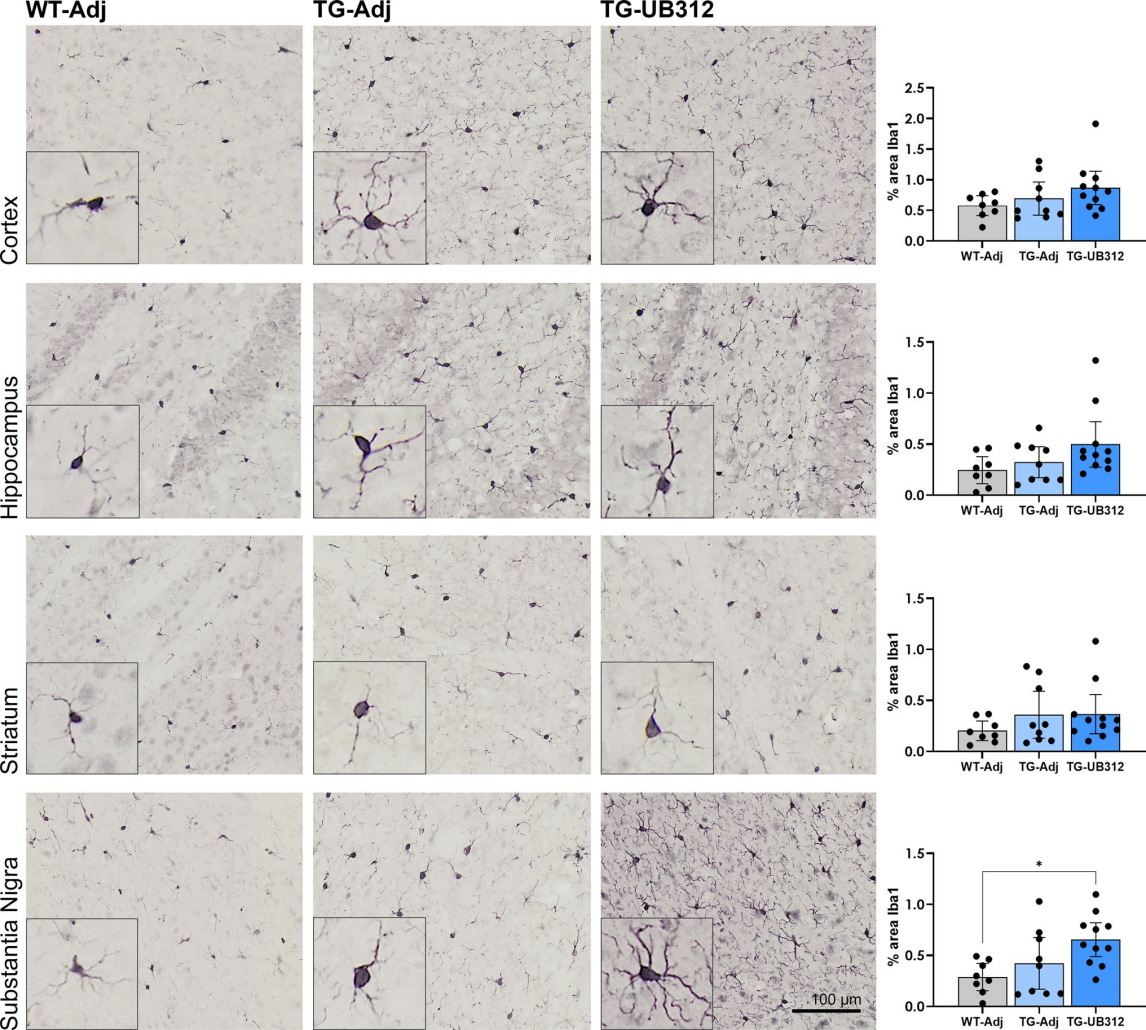
Immunohistochemistry for microglia. Iba1 immunoreactivity was quantified in the cortex, hippocampus, striatum and substantia nigra of Thy1SNCA/15 mice that received UB-312 (*n* = 11) or adjuvant (*n* = 9) or Wt littermates that received adjuvant (*n* = 8). One-way ANOVA with Bonferroni corrections showed a significant increase in the mean percentage area of Iba1 in the substantia nigra. Bars represent mean ± 95% CI

**Fig. 8:**
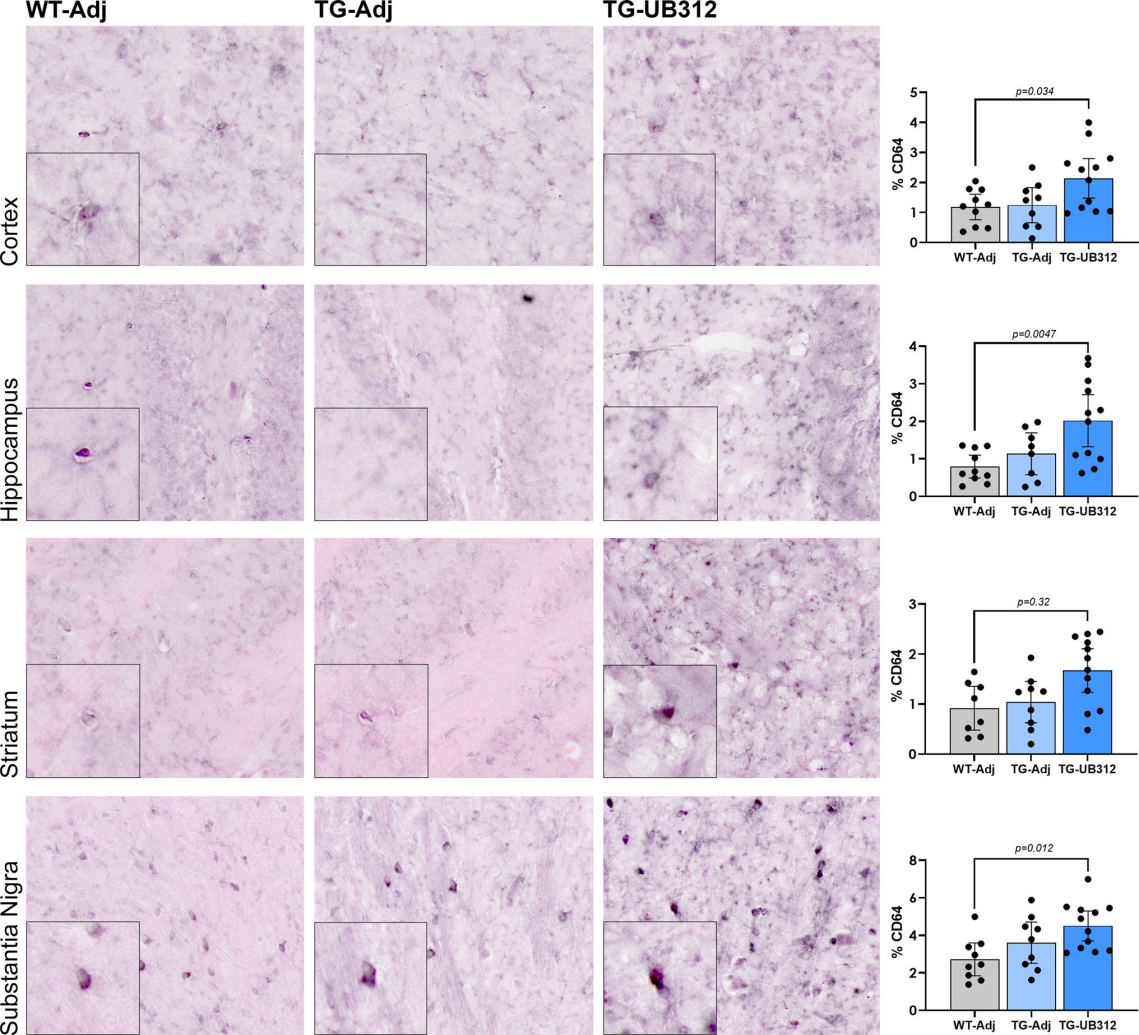
Immunohistochemistry for FcγR1 on microglia. CD64 immunoreactivity was quantified in the cortex, hippocampus, striatum and substantia nigra of Thy1SNCA/15 mice that received UB-312 (*n* = 11) or adjuvant (*n* = 9) or Wt littermates that received adjuvant (*n* = 8). One-way ANOVA with Bonferroni corrections showed a significant increase in the mean percentage area of CD64 after UB-312 immunotherapy in all brain regions analysed. Bars represent mean ± 95% CI

Immunohistochemistry was performed on adjacent tissue sections for markers microglia (Iba1 and CD64). Figures 7 and 8 show representative images of Iba1 and CD64 immunoreactivity, respectively, in each brain region (cortex, Hippocampus, Striatum, Substantia nigra). One-way ANOVA analysis of Iba1 immunoreactivity showed no difference between groups in most brain regions, with the exception of the SN (*F*_(2,25)_ = 4.989) which showed significantly increased Iba1 immunostaining in UB-312 treated Thy1SNCA/15 mice compared to control adjuvant-treated Wt mice (Wt-Adj: 0.29%, Tg-UB-312: 0.65%; *p* = 0.015). Iba1 immunoreactivity was comparable between adjuvant-treated Thy1SNCA/15 mice and Wt mice across all brain regions.

One-way ANOVA analysis of CD64 immunoreactivity showed a significant treatment effect in the cortex(*F*_(2,28)_ = 4.493), hippocampus (*F*_(2,27)_ = 6.652), striatum (*F*_(2,26)_ = 4.700) and substantia nigra (*F*_(2,27)_ = 5.015). Figure 7 shows that UB-312 immunotherapy in Thy1SNCA/15 mice resulted in a significant increase in % area of CD64 immunoreactivity when compared to Wt controls in the cortex by 80% (*p* = 0.039), hippocampus by 155% (*p* = 0.0047), striatum by 82% (*p* = 0.032) and substantia nigra by 65% (*p* = 0.012). CD64 immunoreactivity was comparable between adjuvant-treated Thy1SNCA/15 mice and Wt mice across all brain regions.

**Fig. 9:**
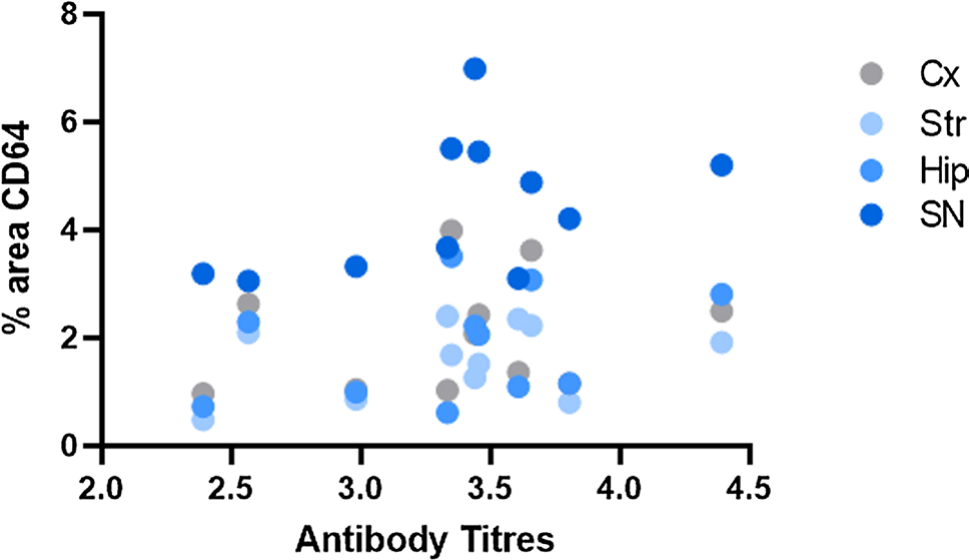
Correlation analysis between CD64 with antibody titres. The average antibody titres produced over the treatment period was calculated for each mouse and plotted against the corresponding CD64 immunoreactivity. Each brain region is represented by a different colour and showed no correlation in the cortex (Cx, *r* 0.229, *p* = 0.497), striatum (Str, *r* 0.334, *p* = 0.315), hippocampus (Hip, r 0.351, *p* = 0.290) or substantia nigra (SN, *r* 0.489, *p* = 0.127

The FcγR1 (CD64) is a high-affinity receptor for IgG. In order to investigate whether the increased CD64 expression was related to the antibody levels produced by each mouse in response to UB-312, a correlation analysis was performed in each brain region. Correlations between antibody titres and CD64 levels are presented in Fig. 9 and show no significant correlation in the cortex (*r* 0.229, *p* = 0.497), striatum (*r* 0.334, *p* = 0.315), hippocampus (*r* 0.351, *p* = 0.290) or substantia nigra (*r* 0.489, *p* = 0.127).

### UB-312 does not induce T cell infiltration

**Fig. 10:**
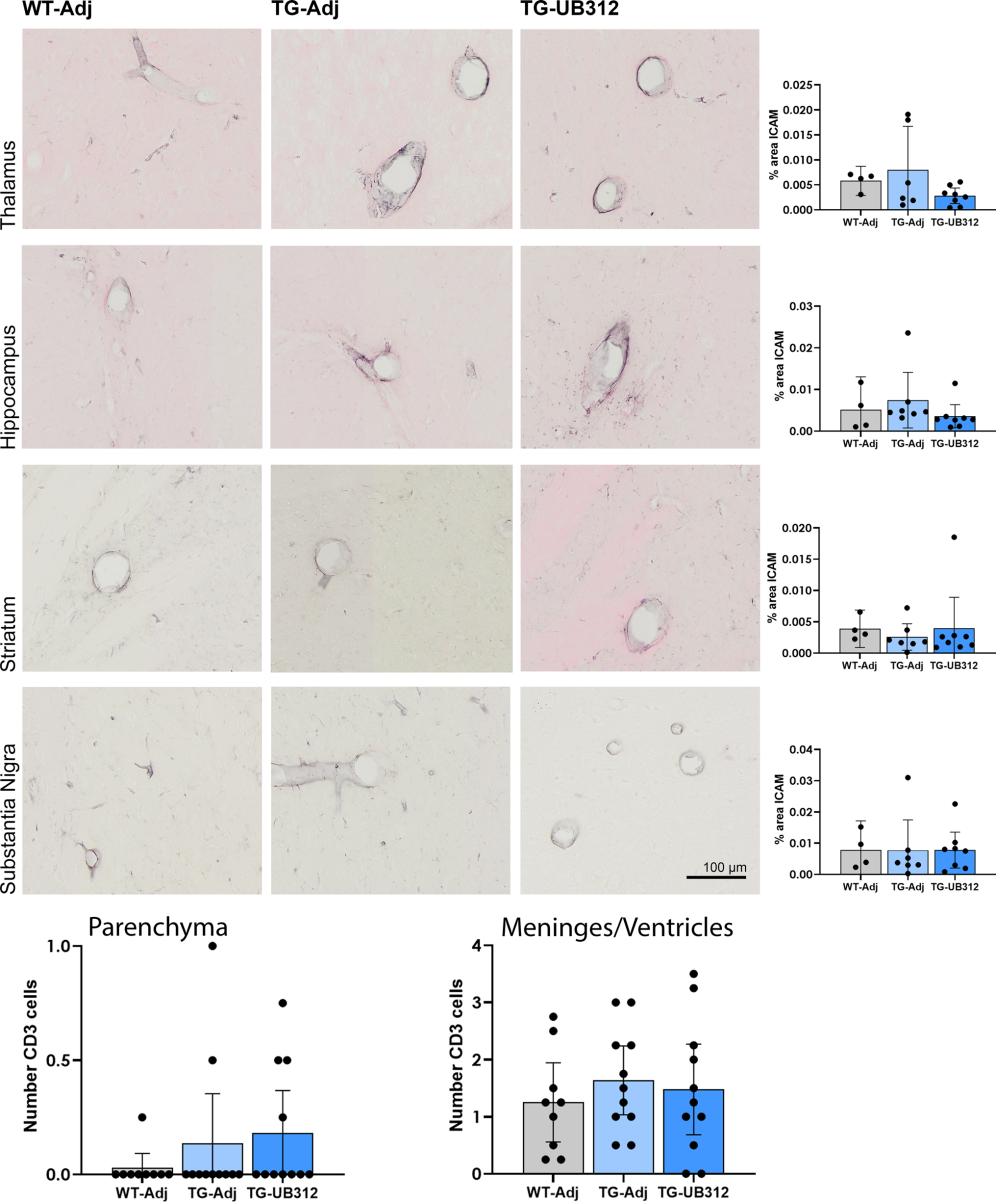
Immunohistochemistry for endothelial activation and T cell infiltration. ICAM1 immunoreactivity was quantified in the cortex, hippocampus, striatum and substantia nigra of Thy1SNCA/15 mice that received UB-312 (*n* = 11) or adjuvant (*n* = 9) or Wt littermates that received adjuvant (*n* = 8). One-way ANOVA showed no difference between treatment groups in each brain region. CD3 + T cells were counted in whole brain sections. Bars represent mean ± 95% CI

The effect of UB-312 treatment on T-cell infiltration was examined by counting the number of parenchymal CD3 positive T-cells over three consecutive 20 µm-thick brain sections. The majority of brain sections were negative for CD3 T-cells and there was no increase in T-cell numbers in UB-312 treated Thy1SNCA/15 mice (Fig. 10). In order to assess the activation state of the endothelia, ICAM1 immunoreactivity on cerebral endothelial cells was quantified. ICAM1 is expressed on endothelial cells and is upregulated during inflammation to facilitate T cell extravasation. The results are presented in Fig. 10 and show no difference in ICAM1 immunoreactivity between UB-312 and adjuvant-treated Thy1SNCA/15 or Wt mice.

### UB-312 reduces αSyn and enteric glial cell activation in the colon

**Fig. 11:**
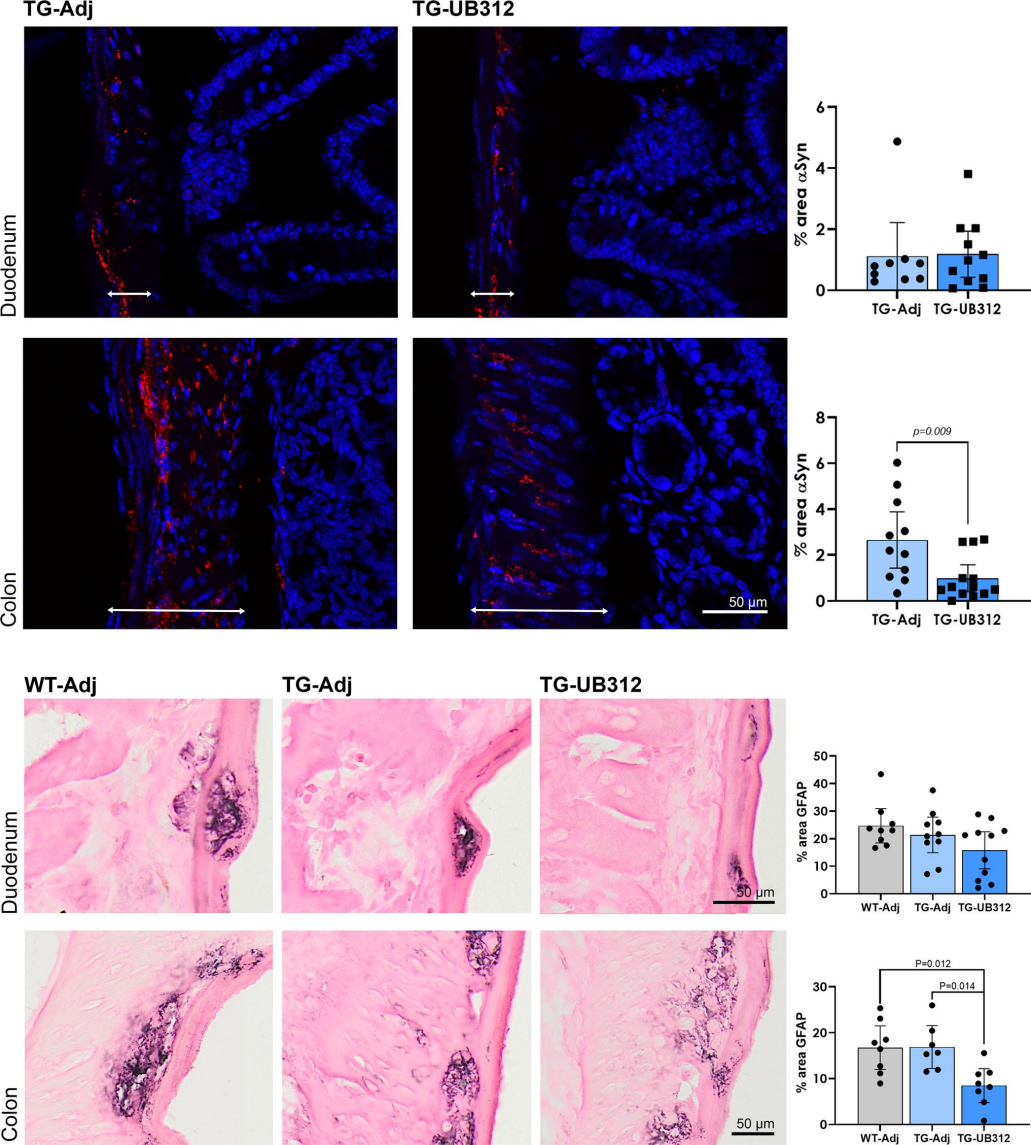
Immunohistochemistry for αSyn and enteric glial cell (EGC) activation in the gastrointestinal tract. Immunofluorescence shows αSyn (red) immunoreactivity in the muscularis (arrows) of the duodenum and colon with DAPI (blue) counterstain. Two-tailed *t*-test showed a significant reduction in the mean percentage area of αSyn in the colon muscularis after UB-312 immunotherapy compared to adjuvant in Thy1SNCA/15 mice. EGC reactivity was measured by quantifying GFAP immunoreactivity in myenteric ganglia. One-way ANOVA showed a significant reduction in the mean percentage area of GFAP in the colon of Thy1SNCA/15 mice receiving UB-312 when compared to adjuvant or Wt littermates receiving adjuvant. There was no effect of UB-312 immunotherapy in the duodenum on either αSyn or GFAP expression levels. Bars represent mean ± 95% CI

Gastrointestinal (GI) dysfunction is a common prodromal feature of PD; and LBs have been identified in colonic biopsies of PD patients. Thy1SNCA/15 mice display αSyn accumulation in the nerve fibres and synapses of the muscularis layer of the gut wall at 10 weeks of age (Fig. 11). Two-tailed *t*-test of the percentage αSyn immunoreactivity in the gut wall showed a significant decrease in UB-312 treated Thy1SNCA/15 mice when compared to adjuvant controls in the colon (Tg-Adj: 2.65%, Tg-UB-312: 0.98%; *p* = 0.0093) but not in the duodenum (Tg-Adj: 1.12%, Tg-UB-312: 1.18%; *p* = 0.91).

The pattern of glial cell reactivity in the gut was investigated using a marker for activation of ganglionic enteric glial cells (GFAP). Figure 11 presents representative images of GFAP immunostaining and subsequent quantification of GFAP immunoreactivity within the myenteric ganglia. One-way ANOVA revealed a significant treatment effect on GFAP expression (*F*_(2,20)_ = 7.007; *p* = 0.0049). UB-312 immunotherapy in Thy1SNCA/15 mice significantly reduced the levels of GFAP expression in the colonic myenteric ganglia when compared to adjuvant-treated Thy1SNCA/15 mice (Tg-Adj: 16.86%, Tg-UB-312: 8.47%; *p* = 0.014).There was no difference in GFAP immunoreactivity between control groups of Wt and Thy1SNCA/15 adjuvant-treated mice (Wt-Adj: 16.73, Tg-Adj: 16.86; *p*  >  0.99), whereas UB-312-treated Thy1SNCA/15 mice showed a significant reduction in GFAP when compared to Wt mice (Wt-Adj: 16.73%, Tg-Adj: 8.47%; *p*  >  0.012). There was no difference in GFAP expression between treatment groups in the duodenum.

## Discussion

UB-312 is a novel active immunotherapy against αSyn developed by Vaxxinity for the treatment of PD patients. After the failure of the first active immunotherapy AN1792 in Alzheimer’s disease, the development of vaccines shifted to favour a passive approach which allows easy termination of the treatment in case of adverse events [35]. However, the relatively short half-life of passive immunotherapies requires monthly infusions of monoclonal antibodies which poses its own problems for diseases that progress over decades. Significant progress has been made in optimizing vaccine technologies that are safe, well tolerated and highly immunogenic. Peptide-based active immunotherapies offer several advantages, these include lower cost of treatment, convenient route of administration (IM) requiring fewer doses, induction of polyclonal antibodies reducing off-target binding compared to monoclonal antibodies and no concern about the anti-drug antibodies induced by some monoclonal antibodies (e.g. Donanemab [35]). Active immunotherapies offer the added advantage of a safe and well tolerated therapy that could be used to prevent the disease years before symptoms occur.

No reported in vivo studies have explored the effects of UB-312 immunisation in transgenic mouse models as yet. While not displaying aggregates as seen in PD, Thy1SNCA/15 mice express human Wt αSyn and do not involve mutated forms of αSyn that are only found in rare familial cases of PD, so Thy1SNCA/15 mice are more representative of the wider population of human PD. In this study the functional and neuropathological benefit of UB-312 immunotherapy was investigated in the transgenic Thy1SNCA/15 mouse model. The UB-312 immunotherapy was well tolerated with all immunised mice completing the study regime without the occurrence of adverse side effects.

### UB-312 targets and clears αSyn oligomers

In our recent study, antibodies produced by UB-312 immunisation were found to be specific for oligomeric and fibrillary αSyn which are the more toxic forms of αSyn [36]. This is consistent with the reduction in oligomeric, but not monomeric, αSyn observed from native western blots in the brains of UB-312 immunised Thy1SNCA/15 mice (Fig. 4). While oligomeric αSyn was reduced by 28–50%, there was only a non-significant trend in decreased monomeric αSyn from the analysis of native western blots. This indicates that the αSyn oligomers are not disassembled into monomers by UB-312 induced antibodies, but are likely being cleared from the brain (discussed further in “Inflammation” section). Although the direct entry of antibodies into the brain was not measured in this study, the reduction in oligomeric αSyn supports that UB-312 immunotherapy has a positive effect on the overall clearance of toxic αSyn species. Thy1SNCA/15 mice overexpress human αSyn and in the present study antibodies specific for human αSyn were used to measure αSyn levels, therefore the effect of UB-312 on endogenous mouse αSyn was not investigated here.

Most studies using Thy1 mice for immunotherapy have used Line 61 mice, whereas the present study used Line 15 mice, which have been reported in the literature only once and demonstrate similar pathology to that described here [8]. The Line15 mice express widespread αSyn in the first few weeks and did not demonstrate LB-like pathology or phosphorylated αSyn at any time point. This is more reminiscent of early stages of PD pathology with elevated oligomeric αSyn but not yet forming LB-like aggregates.

### Inflammation

Overt inflammation with T-cell infiltration has been found to be a harmful side effect of some immunotherapies for neurodegenerative diseases, highlighted by the inflammatory results of the AN1792 clinical trials for AD [33]. The UBITh platform used in the development of UB-312 is designed to avoid an overt pro-inflammatory response while at the same time triggering a robust humoral immune response. This is observed in the present study as UB-312 elicited sustained antibody titres over the course of the treatment period (Fig. 2), but parenchymal T-cell infiltration was not observed (Fig. 10). T-cells were sparse in the brain sections analysed. There was no evidence of endothelial ICAM1 upregulation to facilitate extravasation of T-cells into the brain, supporting the observation of the lack of parenchymal T-cells (Fig. 10).

In neurodegenerative diseases, astrocytes are involved in the clearance of waste products that result from synaptic activity and have been found to accumulate αSyn in synucleinopathies [5, 48, 49]. Astrogliosis is not a consistent observation in synucleinopathies, with conflicting results reported in immunohistochemical studies in both PD and MSA [38, 45, 53]. This study showed no effect of αSyn overexpression on GFAP expression in Thy1SNCA/15 mice at 25 weeks of age and GFAP expression was not affected by UB-312 immunisation. The effect of age and advanced disease stage on astrocyte activation in Thy1SNCA/15 mice would require further investigation and was beyond the scope of the present study. These results are consistent with previous experiments of active immunotherapy in mice overexpressing human-αSyn under the PDGFβ promoter which similarly showed no effect of immunisation on glial cell activation [29].

Although αSyn is a direct, potent activator and chemoattractant of microglia [18, 61], Thy1SNCA/15 mice did not show widespread increased expression of Iba1 in the brain, except in the SN. This may be because these mice were analysed at a relatively early stage in disease pathology and microgliosis may occur with increasing age and advancement of the disease phenotype [8]. This is supported by a recent study in Thy1SNCA/15 mice that showed microglial activation at 10 months of age, but absence of dopaminergic neurodegeneration [8]. After 15 weeks of UB-312 immunotherapy, there was no evidence of widespread glial cell reactivity from microglia or astrocytes, except a slight increase in microglial Iba1 in the SN. Iba1 is a marker of microglial motility and has also been found to be upregulated when microglia become activated. Compared to other brain regions, the SN has been found to contain a higher number of microglia [11, 24, 64] and they are also more sensitive to activation and insult [23, 64]. This may explain the susceptibility of SN to microglial reactivity and the small amount of activation seen only in this brain region after UB-312 immunisation. This study has used a classic method of quantification of immunoreactivity of specific cell markers and while this is a first step in detecting anatomical abnormalities it does not directly inform on morphological changes of specific cells. Microglia are highly motile cells that respond to changes in their microenvironment which can be visualised as a transition from ramified to a more amoeboid morphology during activation [34, 50]. While there were no obvious changes in the morphology or numbers of the cells analysed in this study, further investigation using serology and morphometric analysis approaches would be of interest to fully understand the cellular response to immunotherapy.

While morphological changes in microglia provide information on their general motility and activation state, they do not always correspond to changes in function. In order to investigate this further the expression levels of CD64 were quantified. CD64 is an Fcγ receptor 1, expressed on microglia and macrophages, that has high affinity for IgGs and promotes an activating phenotype [1]. In this study, CD64 was increased after UB-312 immunisation suggesting a possible mechanism of Fc-mediated phagocytosis and clearance of αSyn oligomers bound to IgGs. While the precise mechanism of antibody mediated clearance of αSyn has not been fully investigated in this study, the hypothesised Fc-dependent mechanism is supported by cell culture studies showing that inhibition of Fcγ receptors prevents the antibody-induced clearance of αSyn oligomers [2, 26].

The observation that CD64 expression was not altered between adjuvant treated Wt and Thy1SNCA/15 mice is consistent with post-mortem observations from DLB brains which also show no difference in CD64 levels when compared to controls [1]. However, one study has shown increased CD64 expression in post-mortem PD cases but this was associated with activated amoeboid-shaped microglia [39]. While this study has shown an immunotherapy dependent increase in CD64 expression levels, an important consideration is that there are six Fcγ receptors that differ both structurally and in the downstream signalling cascades that they trigger. The majority of Fcγ receptors induce microglial activation, with FcγRI (CD64) demonstrating the highest affinity for IgGs; however, FcγRIIb induces an inhibitory effect on microglia. To determine the precise effect of immunotherapy on Fcγ receptor expression and microglial function, both inhibitory as well as activating Fcγ receptor expression should be further analysed.

### Behaviour improvement correlates with reduction in brain αSyn oligomers

The line 61 mice have previously shown impairment in beam and wire tests as early as 1–2 months of age [19, 44], and the pole test at 3–4 months [19]. In the current study, line 15 mice demonstrated normal motor performance at 2 months of age and significant deficits at 6 months of age. Thus, immunisation with UB-312 occurred prior to functional changes and prevented the decline in muscle strength (wire test) and co-ordination (beam test). The impairment in motor performance observed in 6-month-old Thy1SNCA/15 mice was likely not a result of neurodegeneration in nigro-striatal pathways as recent studies by Choi et al. demonstrate that Thy1SNCA/15 show no loss of the TH immunoreactivity (marker of dopaminergic degeneration) at 12 months of age [8]. Given that the only observed pathological marker in this study was the increase in oligomeric αSyn species without glial cell activation it is likely that the behavioural improvements are a mediated through neutralization of toxic soluble oligomeric species of αSyn by the anti-αSyn antibodies reaching the brain. The disruption of normal synaptic and neuronal functions by oligomeric species of aSyn has been well described in the literature [3, 12, 41], which supports our hypothesis that neutralizing these toxic species of aSyn should restore normal cellular functions.

The functional improvement is consistent with the reduction in αSyn oligomers observed in basal ganglia regions (striatum and SN). Similar functional improvement has been observed in the wire test for Thy1SNCA/61 mice administered the PD03A active immunisation [28]. Given that Thy1 is a non-specific neuronal promoter, αSyn is likely also expressed in the peripheral motor fibres innervating the muscle which may influence function on some motor tests such as the wire test for muscle strength. This has not been investigated and would require further experimentation to confirm whether this would be a pathological feature.

### The gastrointestinal system

The GI tract does not have a blood brain barrier (BBB) and demonstrated a greater effect of UB-312 treatment on αSyn clearance when compared to the brain that could be observed at the immunohistochemistry level, without the separation of different αSyn assemblies. This indicated that UB-312 has a greater effect on clearing αSyn in the gut when compared to the brain. This is not surprising as the GI tract represents the largest compartment of the immune system containing 70% of the total lymphoid tissue in the body [54]. Our present study showed an effect of UB-312 on αSyn and GFAP expression in the colon but not in the duodenum. This may be because the colon is known to have a higher density of plasma cells compared to other regions of the GI tract making it more responsive to immunotherapy [31]. In the Thy1SNCA/15 mouse model of PD, αSyn expression is directed to neurons and due to the thicker muscularis layer in the colon it is likely that a more marked effect will be seen in the colon.

In this study, the effect of αSyn reduction on colonic function was not assessed; however, other studies using Thy1SNCA/15 mice have shown that αSyn expression in the gut contributes to GI dysmotility [7, 60]. Interestingly, Squalamine, an anti-cancer drug isolated from the liver of the dogfish shark, has been found to prevent the formation of αSyn oligomers and aggregates, and consequently improve colonic motility in A53T mouse models of PD [62]. This suggests that oligomeric αSyn plays a role in gut dysmotility and suppression of their toxic effects improves GI function. This is consistent with the findings of the present study suggesting that UB-312 targets and facilitates the clearance of oligomeric αSyn.

The effect of UB-312 on enteric glial cell (EGC) activation was assessed by immunohistochemistry for GFAP. EGCs play a vital role in maintaining homeostasis and neuronal function. Dysfunction in EGCs has been associated with loss of neurons an subsequent impaired gut functions [56]. EGC activation has been found in colonic biopsies from PD patients, which have shown upregulation of GFAP when compared to healthy controls [9]. This has also been replicated in a rotenone mouse model of PD that has found a correlative pattern in enteric αSyn expression and EGC activation [14]. Here, together with the reduction in αSyn, UB-312 immunotherapy caused a decrease in GFAP expression in the myenteric ganglia of the colon. This was not observed in the duodenum, which only showed a trend towards decreased GFAP expression after UB-312 treatment, correlating with the lack of treatment effect on αSyn pathology. Together, this suggests that reduction in αSyn oligomers has a neuroprotective effect in the gut to preserve neuronal function; however, further investigation into the functional state of neurons with electrophysiology would be required to confirm this hypothesis.

## Conclusion

UB-312 was well tolerated in Thy1SNCA/15 mice with improved functional performance and reduced pathology in both the brain and colon. This is the first immunotherapy in clinical trials to our knowledge that has demonstrated improved αSyn pathology in the GI tract. Further investigation would be required to analyse whether this corresponds to improvement in GI function. Our study demonstrates the potential for UB-312 to treat PD cases at the earliest stages of the disease and possibly prevent the progression of both motor and non-motor symptoms, although this strategy would also require improved biomarkers for distinguishing PD at its early stages.

The effect in behaviour and neuropathology with UB-312 treatment is relatively small, although still significant, and may be due to the limited access of antibodies to the brain. The penetration of UB-312 generated antibodies into the brain was not directly investigated in this study but other studies have shown that less than 1% of peripheral antibodies enter the brain [6]. In light of this, the lack of a BBB in the GI tract may explain the pronounced reduction in αSyn with UB-312 immunotherapy. In addition, given that Thy1SNCA/15 mice overexpress human-αSyn, the increased rate of production of αSyn may also counteract the rate of clearance facilitated by UB-312, whereas neurodegenerative diseases are known to be chronic, progressive diseases that accumulate protein over decades.

PD patients at clinical presentation already have substantial αSyn pathology in their brains and this study has demonstrated the potential for UB-312 immunotherapy to have a beneficial functional and neuropathological effect after αSyn accumulation has commenced, as well as have the potential to treat non-motor symptoms of PD.

## Supplementary Information

Below is the link to the electronic supplementary material.

Supplementary file1 (TIFF 9471 KB)
