# Supplementary figures and images for: Immunisation with UB-312 in the Thy1SNCA mouse prevents motor performance deficits and oligomeric α-synuclein accumulation in the brain and gut
Source: Acta Neuropathol. 2021 Nov 6;143(1):55–73. doi: 10.1007/s00401-021-02381-5 (PMC8732825; doi:10.1007/s00401-021-02381-5)

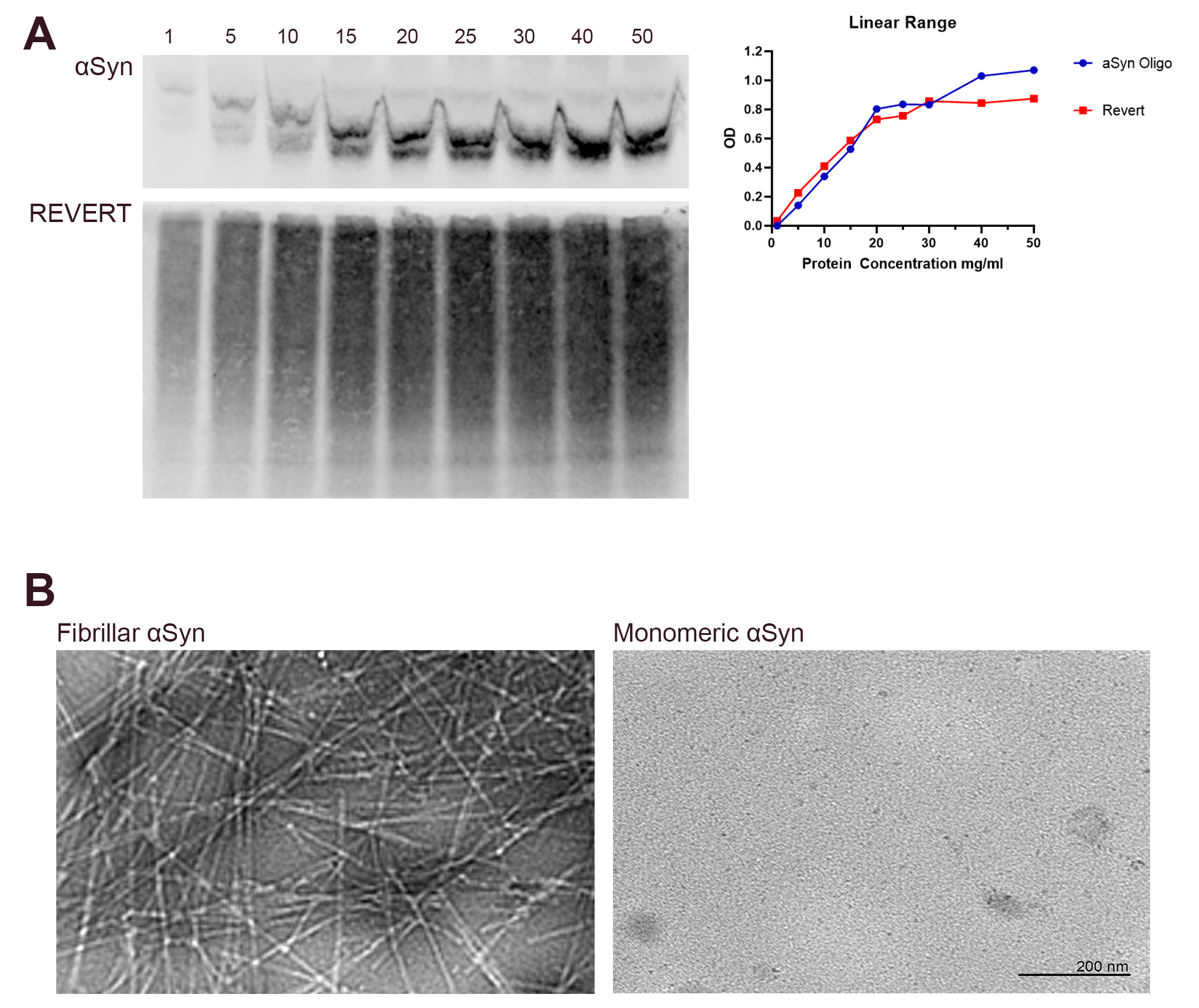

Supplement: Supplementary file 1 — Supplementary file1 (TIFF 9471 KB) [file 401_2021_2381_MOESM1_ESM.tiff]
